# First-Principles Band Alignments at the Si:Anatase TiO_2_ Interface

**DOI:** 10.1021/acsomega.3c02865

**Published:** 2023-05-22

**Authors:** Yide Chang, Jonathan R. Yates, Christopher E. Patrick

**Affiliations:** Department of Materials, University of Oxford, Parks Road, Oxford OX1 3PH, United Kingdom

## Abstract

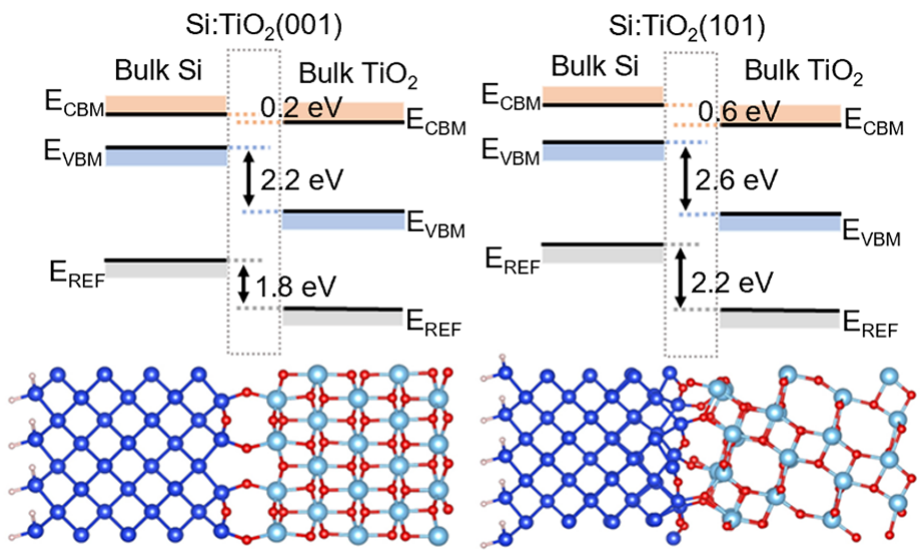

TiO_2_ has been identified as a promising electron transport layer in Si solar cells. Experiments have revealed that the Si:TiO_2_ interface undergoes structural changes depending on how it was fabricated. However, less is understood about the sensitivity of electronic properties, such as band alignments, to these changes. Here, we present first-principles calculations of band alignments between Si and anatase TiO_2_, investigating different surface orientations and terminations. By calculating vacuum-level alignments, we observe a large band offset reduction of 2.5 eV for the O-terminated Si slab compared to other terminations. Furthermore, a 0.5 eV increase is found for the anatase (101) surface compared to (001). We compare the band offsets obtained through vacuum alignment with four different heterostructure models. Even though the heterostructure models contain an excess of oxygen, their offsets agree well with vacuum-level alignments using stoichiometric or H-terminated slabs, and the reduction in band offsets seen for the O-terminated Si slab is not observed. Additionally, we have investigated different exchange-correlation treatments including PBE + U, postprocessing *GW* corrections, and the meta-GGA rSCAN functional. We find that rSCAN provides more accurate band offsets than PBE, but further corrections are still required to achieve <0.5 eV accuracy. Overall, our study quantifies the importance of surface termination and orientation for this interface.

## 1 Introduction

Despite significant and rapid developments in alternative designs and materials, the commercial manufacture and distribution of photovoltaic (PV) cells remains dominated by crystalline silicon (c-Si) devices,^1^ with passivated emitter and rear contact (PERC) cells estimated to account for 90% of the global market share in 2021.^2^ The PERC design was developed in part to address a performance bottleneck occurring at the contacts between the light-absorbing c-Si and the metal electrodes connecting the device to the external circuit, by employing “local” contacts which minimize the surface area of the metal–semiconductor interface.^3^ In an alternative design to PERC, passivating contact cells^4^ attempt to overcome this bottleneck by removing all direct contact between the light absorber and metal terminal, by inserting a thin layer of another material between them. This approach carries the dual advantage of reducing the number of surface states (by passivating c-Si dangling bonds) and also increasing the physical separation between the metal and Si, which weakens the Fermi level pinning.^5^ Furthermore, depending on the alignment of the electronic energy levels at the interface, the passivating layer can form a carrier selective contact, whereby only one type of charge carrier (electrons or holes) is able to pass through the passivating layer, greatly reducing recombination losses at the electrode.^6^

Passivating contact cells employing amorphous Si or hydrogenated microcrystalline silicon have achieved power conversion efficiencies in excess of 25% .^7,8^ However, the enhanced performance can come with an increased manufacturing cost, in particular if toxic gas precursors are required in the fabrication process.^9^ In this respect, transition metal oxides (TMOs), which may be deposited using lower-cost techniques, are attractive alternative passivating materials.^10^ The diversity of electronic and optical properties within the TMOs (and the potential to tune these properties through control of structure and stoichiometry) raises the prospect of designing interfaces which can provide optimal carrier transport simultaneously with favorable optical properties,^11^ as well as mixing-and-matching a number of different TMOs in a single device to obtain electron and hole selectivity at different contacts.^12^

Among the TMOs, TiO_2_ has been identified as a particularly interesting material for passivating contact cells.^9,12−14^ With its large (UV) band gap and Fermi level close to the conduction band, TiO_2_ is expected to form an electron-selective contact, efficiently blocking holes at the interface with c-Si.^10,15^ However, by reducing the oxygen content to form TiO_*x*_ nanolayers, the authors of ref (16). demonstrated the versatility of the TiO_2_ passivating layer by creating *hole*-selective contacts. Apart from stoichiometry, experimental studies have also illustrated relationships between the atomistic structure of the interface and its electronic properties, such as the apparent benefits of amorphous TiO_2_ compared to anatase in forming a defect-free surface.^17,18^

The parameter space associated with synthesizing the Si:TiO_2_ heterojunction is very large, considering the different precursors, the deposition method, and subsequent annealing treatment. Systematic variation of the different parameters, followed by detailed characterization at the material and device level, is required to identify optimal routes to improve the performance of TiO_2_-based passivating contact cells, but realizing such studies experimentally places high demands on researcher time and resources. First-principles calculations provide a complementary means of investigating the interface, particularly its fundamental electronic properties. Despite the huge importance of both Si and TiO_2_ as functional materials, first-principles modeling of their interfacial properties is a relatively unexplored area, not least because of the computational and conceptual challenges associated with the aforementioned large parameter space.

Here, we focus on a particular quantity—the interfacial band alignments, which are crucial to determining the carrier selectivity as well as device characteristics like fill factor and open-circuit voltage^19^—and report a computational study based on density-functional theory (DFT) into their dependence on the atomistic structure of the interface. Choosing Si(100) and anatase TiO_2_ as our prototype system, we first consider the materials in isolation and calculate the band edge positions with respect to the vacuum. Applying a common vacuum level across the materials provides a first estimate of the band alignments, but neglects any effects of the redistribution of charge when the interface is formed. Accordingly, we then consider candidate interface models which vary in terms of surface orientation, the type and number of bonds formed, and strain across the interface. Calculating the conduction and valence band offsets directly for these models allows us to quantify the influence of the above factors. Concurrently, we have studied the importance of going beyond standard generalized-gradient approximations (GGAs) for the exchange-correlation energy in DFT when calculating the band alignments, in particular investigating the relatively new meta-GGA rSCAN functional.^20^ As such, our work provides a fundamental view of this important interface, and also helps determine a computational strategy for future modeling of interfaces in TMO-based cells.

## 2 Methods

### 2.1 Calculating Band Alignments

Band alignments are calculated using the established approach as described, e.g., in ref (21). First, calculations on bulk materials are used to determine the electronic energy levels with respect to an average reference potential. Then, calculations are performed in slab geometries either to determine the position of the same reference potential with respect to the vacuum^22^ or to determine its variation across the heterointerface.^21^ In the following we refer to the first approach as “vacuum-level alignment” and the second as “heterointerface alignment”. As our reference, we use the planar-averaged Kohn–Sham potential, as extracted by the c2x software package.^23^ We note that the electronic energy levels obtained in DFT (Kohn–Sham eigenvalues) do not formally correspond to the fundamental quantities which determine band alignments, i.e., the quasiparticle addition and removal energies.^24^ Accordingly, in our study we investigate the sensitivity of the band alignments to different descriptions of exchange and correlation.

### 2.2 Constructing Surface and Interface Models

Considering Si first, in line with experimental studies on the interface^16,25,26^ we focus primarily on the (100) surface. Cleaving c-Si along this plane leaves dangling bonds at the surface. For the vacuum level alignment calculations, we investigated saturating these dangling bonds either through atomic passivation (H or O) or by allowing the surface to undergo a (2 × 2) reconstruction.^27^ For the heterointerface alignment, the TiO_2_ provides under-coordinated O atoms which can be used to saturate some, but not all, of the bonds. Following an approach employed in ref (22) to construct (100)Si:ZrO_2_ interfaces, we introduced additional oxygen atoms to saturate the dangling bonds, producing an interface which is insulating. The precise structures and stoichiometries are discussed in more detail below.

Modeling the TiO_2_ side of the interface presents a more difficult challenge. TiO_2_ exists naturally in different phases, most commonly rutile and anatase,^28^ but experimental studies on Si:TiO_2_ have targeted the production of amorphous TiO_2_ due to its apparently superior passivation qualities.^9^ Specifically, the presence of anatase was linked to increased recombination at the Si:TiO_2_ interface, which in turn was attributed to the crystalline phase increasing stress and generating defects.^29^ However, anatase is the most commonly found form of TiO_2_ at the nanoscale,^28^ and refs (17 and 18) reported that the anatase phase will be present in Si:TiO_2_ interfaces if the deposition temperature is of order 300 °C, with lower temperatures required to form an amorphous layer. Seeing that its bulk crystal and electronic structures have been studied in detail previously,^30−32^ we have chosen to use anatase as the basis of our Si:TiO_2_ interface models. We have investigated the two lowest-energy anatase surfaces, (101) and (001).^28,33^ Both surfaces are stoichiometric and insulating but nonetheless have under-coordinated Ti and O atoms which are available to form bonds.^34^

The surface and interface models were constructed using periodically repeated simulation cells. We first performed geometry optimizations of the bulk crystal structure, investigating different exchange-correlation functionals, and then copied and cleaved the relaxed bulk structures to obtain slab models. These slab models were then reoptimized (with cell dimensions held fixed according to their bulk values). All optimized structural slab models are provided as supplementary data.

For vacuum level alignments, for Si we used a (100) slab of approximate lateral dimensions 16 Å × 8 Å (8 surface atoms) and thickness 11 Å (9 atomic layers), with a vacuum region of 20 Å separating periodic replicas along the surface normal. We investigated models where both the top and bottom surface underwent a (2 × 2) reconstruction, and also where the dangling bonds on the lower surface were saturated with H, and the bonds on the upper surface saturated either with O (OH) or H (HH). We also investigated a reconstructed Si(111) slab with dimensions 4 Å × 13 Å (4 surface atoms) and thickness 20 Å (14 atomic layers), with a vacuum region of 20 Å separating periodic replicas along the surface normal. For TiO_2_ we investigated stoichiometric (101) and (001) slabs of lateral dimensions 10 Å × 4 Å and 4 Å × 4 Å with thicknesses of 13 and 20 Å respectively, and a vacuum region of 23 Å.

**Figure 1:**
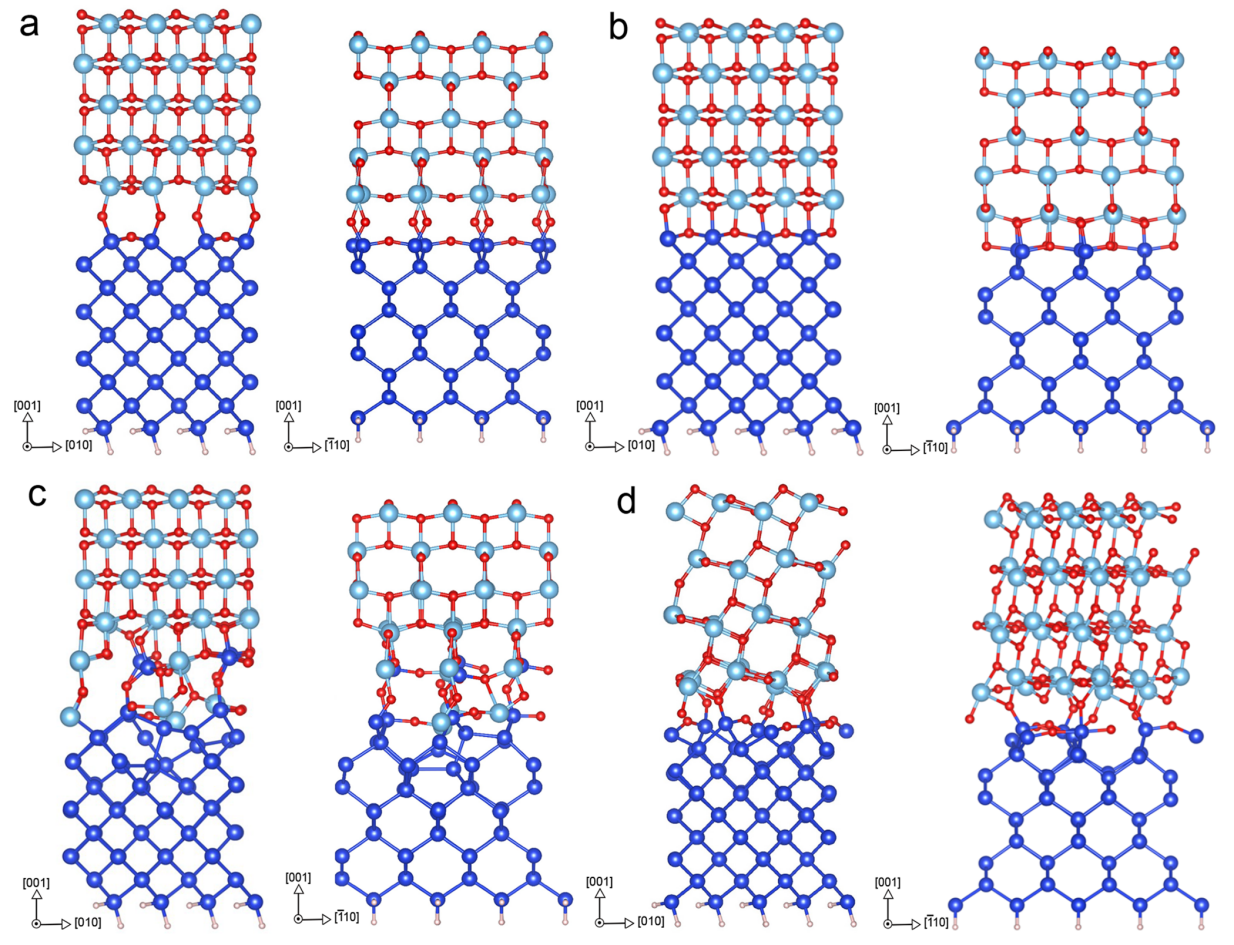
Atomic structure of (a) Model 1, (b) Model 2, (c) amorphous Si:TiO_2_(001), Model 3, and (d) Si:TiO_2_(101), Model 4, interface models. Si/H/O/Ti atoms are shown in blue/pink/red/light blue, respectively.

For the heterointerface alignment, we have focused on four fundamental models of the interface, shown in Figure 1 and discussed further in Section 3.5. All are based on Si(100) with a monolayer coverage of O atoms corresponding to one additional O atom per surface Si atom, which saturates the dangling bonds.^22^ Models 1 and 2 are both highly ordered interfaces formed with the anatase (001) surface. Model 3 is also based on the anatase (001) surface, with a series of atom swaps followed by first-principles molecular dynamics used to create a disordered interface. Model 4 is another highly ordered interface but formed with the anatase (101) surface.

The majority of the heterointerface alignment calculations were carried out in a “single interface” arrangement, which corresponded to an 11 Å-thick slab (9 layers) of Si and a 10 Å-thick slab of TiO_2_, followed by 20 Å of vacuum. The dangling bonds on the lower face of the Si slab were saturated with H atoms. Furthermore, for the majority of calculations the cells were constructed using the relaxed Si lattice parameters, with lateral dimensions of the unit cell of 6 Å × 6 Å for models 1–4. As discussed in Section 3.5, we also investigated cells constructed using the TiO_2_ lattice parameters.

### 2.3 Computational Details

All DFT calculations on the Si:TiO_2_ interface were performed using plane-wave basis sets, periodic boundary conditions, and ultrasoft pseudopotentials^35^ as implemented in the CASTEP package.^36^ Exchange and correlation effects were included either at the GGA level using the PBE functional,^37^ at the GGA+*U* level (also PBE),^38^ or using the meta-GGA rSCAN functional.^20^ The maximum cutoff energies for the plane wave basis sets were 700 eV for PBE and PBE+*U* and 800 eV for rSCAN calculations. Spurious interactions between periodic slab replicas were minimized through use of the large vacuum region and the self-consistent dipole correction.^39^ Reciprocal space sampling was performed on Monkhorst–Pack grids^40^ with dimensions 6 × 6 × 1 for vacuum level alignment and 4 × 4 × 1 for single interface, or 8 × 8 × 8 for bulk. Geometry optimizations were carried out until the force on each atom was below 0.05 eV Å^–1^, and for the bulk calculations, the convergence threshold for pressure was 0.1 GPa. In the generation of Model 3 we carried out several picoseconds of Car–Parrinello molecular dynamics as implemented in the cp.x program which forms part of the Quantum ESPRESSO distribution, using a Nosé-Hoover thermostat.^41,42^ This structure was subsequently fed back into CASTEP and reoptimized. We stress that this procedure was solely carried out to produce a disordered interface structure, and is not intended to be a realistic representation of annealing.

## 3 Results

### 3.1 Review of Experimental Measurements of the Si:TiO_2_ Band Alignments

Various experimental studies^25,26,43^ have quoted previously published measurements of the band edges of various forms of isolated TiO_2_, with the conduction and valence bands at ∼4 eV and 7–7.2 eV below the vacuum level, respectively, and 4.05 and 5.17 eV for the conduction and valence bands of Si(100). Aligning to a common vacuum level gives approximate conduction and valence band offsets (CBOs and VBOs) of 0 and 2 eV, respectively. The CBO of 0 eV implies the conduction bands are degenerate, allowing free movement of electrons across the interface, but the sizable VBO acts to prevent holes from traveling from Si to TiO_2_. The device characteristics measured for the Si:TiO_2_ interfaces fabricated in these works were found to be consistent with this qualitative picture of an electron-selective contact. Interestingly, very similar offsets (0 and 1.95 eV) are predicted based on the alignment of the charge neutrality levels of the two materials.^44,45^ Also, it should be noted that there is substantial variation in reported band edge positions for TiO_2_, which display a high sensitivity to preparation conditions; in ref (46), valence band positions of 7 eV below vacuum corresponded to ex-situ preparation.

We are also aware of two studies which measured directly the band alignments at the interface. Direct and inverse photoemission spectroscopy (PES) reported in ref (14) yielded a CBO with a magnitude of less than 0.3 eV and a VBO of ≥2.3 eV for Si:TiO_2_. Reference (19) also reported direct PES measurements, of Si:TiO_*x*_ (*x* = 2.02, i.e., slightly oxygen rich), and found a reduced VBO, of ∼1.7 eV.^10^ For later discussion, it is important to note that these works predict the conduction band to lie higher in energy for TiO_2_ than Si, thus forming a type I heterojunction. However, ref (47) reported the fabrication and band alignments of Si:IL:TiO_2_ interfaces, where the interlayers (IL) investigated were Al_2_O_3_, Si_3_N_4_, and Ti-rich SiO. Here VBOs of 2.4–2.7 eV and CBOs of 0.2–0.5 eV were reported. In these cases the Si conduction band lay higher in energy than TiO_2_, forming a type II heterojunction.

### 3.2 Vacuum Level Alignments and Effects of Surface Termination

**Figure 2:**
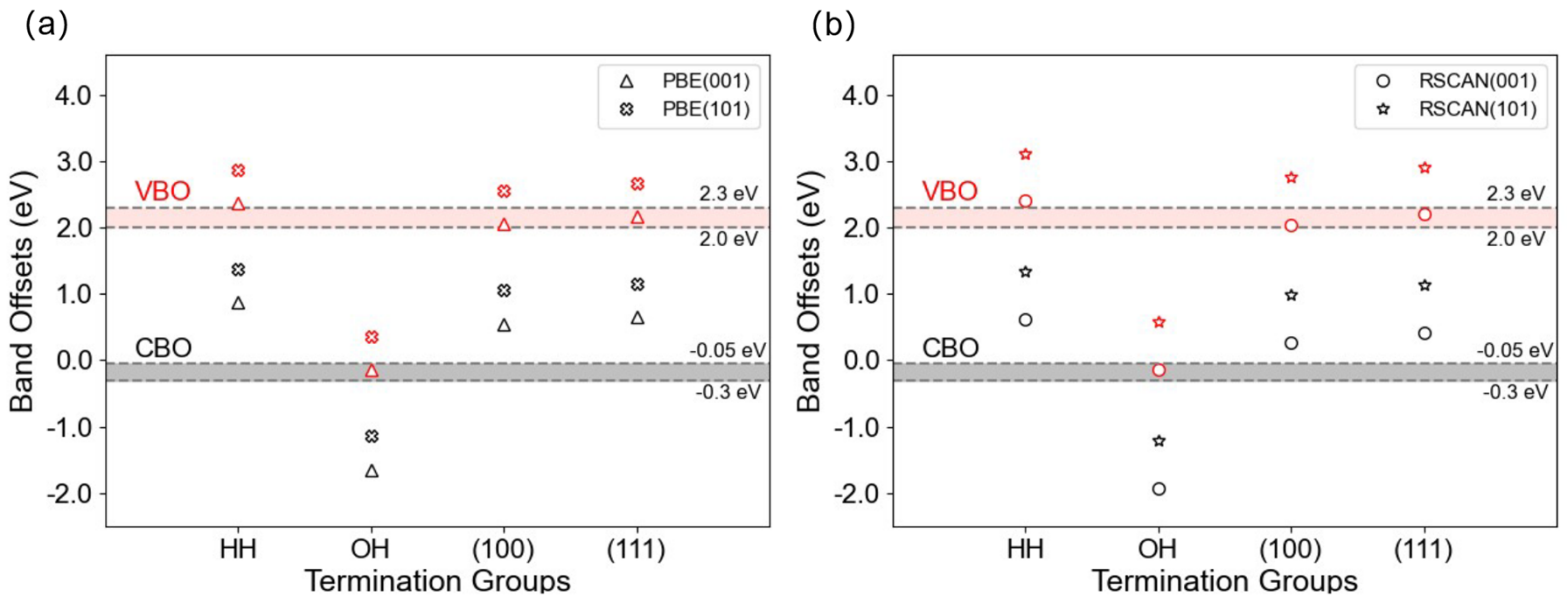
Calculated VBOs, shown in red, and CBOs, shown in black, of Si:TiO_2_(001) and Si:TiO_2_(101) interfaces, using (a) PBE and (b) rSCAN. The shaded regions denote the range of values previously reported in experimental literature, as discussed in Sec. 3.1.

Figure 2 shows the valence and conduction band offsets calculated for the Si:TiO_2_ interface assuming vacuum-level alignment. The offsets are presented for different surface terminations of the Si(100) surface and the pure (100) and (111) reconstructions, for the stoichiometric anatase (001) and (101) surfaces. The figure also compares the offsets calculated with the PBE GGA and the rSCAN meta-GGA exchange-correlation functionals. The raw ionization potential and electron affinity (IP and EA) data used to construct these offsets is given as Supporting Information.

The most dramatic feature of Figure 2 is the 2–2.5 eV reduction in band offsets found for the oxygenated Si slab, compared to the H termination or pure reconstruction. This is due to a large increase in the IP and EAs for O termination. By contrast, the IP and EA are shifted in the opposite direction for H termination, compared to the pure reconstruction, albeit by a much smaller amount. This behavior mirrors that found for the diamond C(100) surface, as studied in ref (48). However, unlike for diamond, we found the most stable oxygenated Si(100) surface to comprise a mix of “bridge” and “ketone” adsorption modes, arranged in a 2 × 2 reconstruction. We checked the stability of this reconstruction for the diamond C surface and found that the surface relaxed to a pure “bridge” structure, in line with previous results.^48^ We attribute this difference between the silicon and diamond surfaces to the increased Si–Si bond length compared to C.

Comparing the circles and stars or triangles and crosses in Figure 2 also shows how more positive band offsets are predicted for the anatase (101) surface compared to (001), due to the IP and EA of the former being larger by 0.46 eV. The experimental measurements of ref (46) also found an increased IP for the (101) surface, of 0.1–0.5 eV depending on different surface conditions, which agrees well with our calculated increase. The different offsets calculated for the Si(100) and (111) reconstructions reflect that we calculate a smaller IP and EA for the latter (by ∼0.1 eV), in good agreement with previous calculations.^49^

Comparing the calculated band offsets to those previously reported in experimental literature (shaded in Figure 2), we see the closest agreement for the VBOs with nonoxygenated Si. However, the CBOs for these cases are large and positive, corresponding to the Si conduction band lying higher in energy than the conduction band of TiO_2_. More generally, we note that the calculated IP for TiO_2_ of ∼7 eV is quite close to the values quoted in experimental literature, but the EA of 4.9 eV is too large. This naturally leads us to consider the effect of exchange-correlation treatment, as discussed in Section 3.3.

Overall, the results in Figure 2 emphasize that the band offsets calculated through vacuum alignment can vary substantially depending on how the constituent slabs are terminated. Such terminations are relevant, since experimental devices may involve modifications like oxygen-rich or deficient TiO_2_,^19^ SiO_2_,^9^ or adsorbed H.^16^ On the other hand, one would expect the electric dipoles associated with these terminations to be screened in the full interface, an effect which is missing in the vacuum alignment approach. This motivates our investigation of heterointerface alignments in Section 3.5.

### 3.3 Influence of Exchange-Correlation Treatment

The large values of the calculated EAs reported in the previous section are a manifestation of DFT’s “band gap problem” (Section 2.1). Various schemes can be used to improve the quantitative accuracy of the calculations, including hybrid functionals^50,51^ or many-body perturbation theory in the *GW* approximation.^51,52^ For the case of TiO_2_, the valence and conduction bands are predominantly O-2*p* and Ti-3*d* character, respectively.^32^ Applying a Hubbard *U* correction to the Ti-3*d* orbitals therefore has the effect of opening the band gap.^32,50,53^ The advantage of this GGA+*U* approach is that it is computationally efficient and therefore can be applied to large-scale interface models. It might be particularly important for interfaces where, without any correction applied, the TiO_2_ conduction band would be erroneously placed lower in energy than the valence band of the other material. In this case, the interfacial electronic structure would be qualitatively incorrect, since electrons would populate TiO_2_ conduction states, leading to metallic behavior.^54^ A disadvantage is the numerical freedom associated with the *U* parameter, which can be determined self-consistently^55^ but can also be treated as a variable to be optimized.

**Figure 3:**
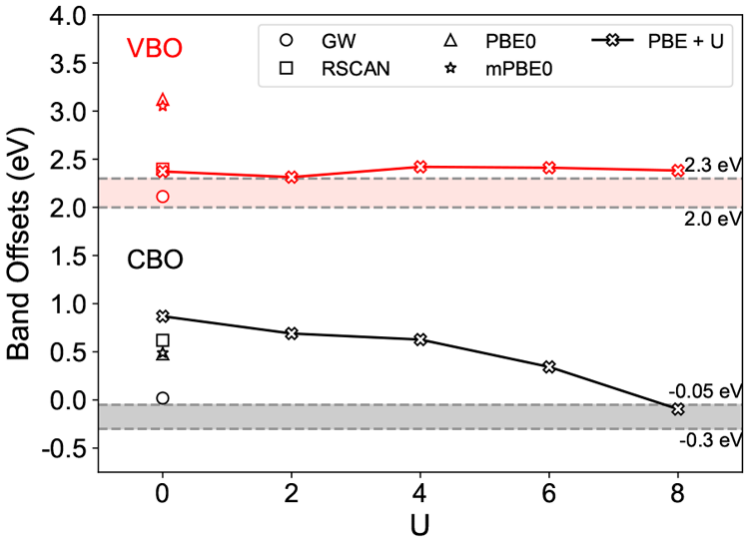
Calculated VBOs and CBOs of the Si-HH:TiO_2_(001) interface using PBE+*U* as the *U* value increases from 0 to 8 eV. Band offsets obtained using corrected band edges from *GW*, mPBE0, and PBE0 are also included.

Figure 3 shows vacuum alignment calculations for the H-terminated (100)Si:TiO_2_(001) interface using the GGA (PBE)+*U* description of exchange and correlation, for values of *U* from 0–8 eV. Over this wide range of *U* values, the calculated VBO is very stable, fluctuating by less than 0.2 eV. Although in some sense this is not surprising since the Ti-3*d* content of the valence band is low, it is important to note that the *U* parameter does also cause a lattice expansion,^32,56^ which might be expected to shift the band edges.

On the other hand, the CBO is very sensitive to the *U* value. Increasing *U* opens the band gap by reducing the EA, with a gap of 3.09 eV obtained for a *U* value of 8 eV. The conduction band is almost entirely composed of Ti-3*d* orbitals, and the *U* correction applied to these orbitals reduces their hybridization with O-2*p* states and raises their energy. It has also been shown previously that the *U* correction reduces the conduction bandwidth by merging together the *t*_2*g*_ and *e*_*g*_-derived subbands.^32^ Therefore, as the *U* correction increases, the TiO_2_ conduction band is raised in energy, and the CBO reduces, since in the absence of *U* corrections the Si conduction band lies higher in energy than the TiO_2_ (Sec. 3.2). Although it is not necessarily straightforward to compare different implementations of GGA+*U*, the quantitative dependence of the gap on *U* value matches well to previous studies.^32,50,53^ In particular we note that values of *U* determined self-consistently (e.g., 2.5 eV in ref (50)) produce gaps much smaller than the (optical) gap of anatase of 3.2 eV.^57^

As an alternative to the fully self-consistent calculations presented so far, we can instead follow the approach, e.g., of refs (52 and 54) and use higher levels of theory to correct the band edge positions, as a postprocessing step. For instance, corrections to the GGA Si valence and conduction bands of −0.4 eV and +0.2 eV were calculated within the *GW* approximation in ref (52), while ref (53) reported corrections of −0.14 eV and +1.05 eV for anatase TiO_2_. For the H-terminated interface considered above, this has the effect of decreasing the VBO by 0.26 to 2.11 eV and the CBO by 0.85 to 0.02 eV. As shown in Figure 3 these values are rather close to the band offsets reported previously in the experimental literature.

We have also investigated corrections derived from hybrid functionals. Reference (58) reported corrections of −0.31 eV and +0.28 eV to the Si valence and conduction band, using a modified (m) PBE0 functional where the exact exchange fraction (α = 0.11) was tuned to produce a band gap of 1.2 eV. Reference (50) also used PBE0 with a modified value of α = 0.18 (tuned to give a self-consistent inverse dielectric constant) to obtain corrections of −0.99 and 0.66 eV for anatase TiO_2_. Altogether this increases the VBO by 0.68 eV and decreases the CBO by 0.38 eV. We also show in Figure 3 the shifts calculated using the standard PBE0 functional with α = 0.25.^50,51^

The spread of values in Figure 3 demonstrates the strong dependence of band offsets (particularly the CBO) on the theoretical approximation used. Although DFT+*U* allows self-consistent calculations to be performed at a computational cost similar to a standard GGA, it is difficult to justify using the *U* value > 8 eV which would be required to reproduce the TiO_2_ band gap. Hybrid functionals come at a much increased computational cost and require a scheme to justify the exchange fraction α. Calculations involving the *GW* method are the most computationally expensive, and in most cases are only feasible as a non-self-consistent, postprocessing step. However, they do also have the strongest theoretical justification, and it is straightforward to apply the corrections since they are a property of the bulk system. It is trivial to obtain *GW*-corrected values of all of the PBE data points in Figure 2 simply by shifting the VBOs and CBOs by −0.26 eV and −0.85 eV, respectively.

### 3.4 rSCAN

Figure 3 also shows how the band offsets calculated with the meta-GGA rSCAN functional lie between the PBE and *GW* values. This result is consistent with a recent study which surveyed a number of meta-GGA functionals^59^ for calculating band alignments, including the closely related r^2^SCAN functional.^60^ As pointed out by the authors of that work, being able to obtain accurate band offsets at the meta-GGA level is a very attractive prospect, since the computational expense is much lower than for hybrid functionals or *GW*.

**Figure 4:**
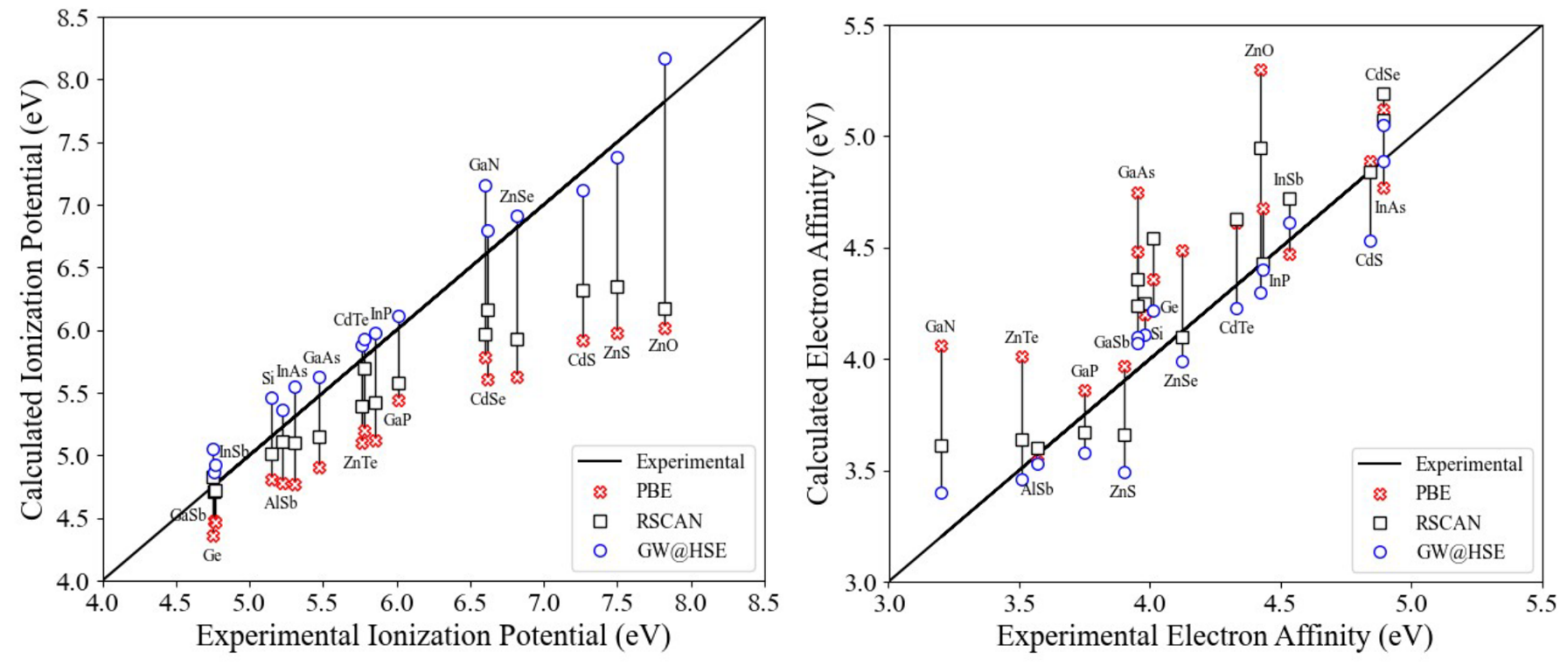
IP and EA of 17 semiconductors obtained using PBE and rSCAN along with reported experimental and *GW@HSE* calculated values.^61^ Computational details of these calculations are included as Supporting Information.

To better understand the performance of rSCAN we have calculated the ionization potentials and electron affinities for a test set of semiconductors, following ref (61). The results are summarized in Figure 4 and demonstrate that rSCAN does systematically improve the description of these quantities compared to PBE, when compared to experiment or *GW* calculations. We note however that inaccuracies remain. The authors of ref (59) have pointed out that since band offsets are based on differences in IPs or EAs, rather than their absolute values, there will be error cancellation when calculating the VBOs and CBOs. Figure 4 shows that agreement between calculated and experimental IPs systematically worsens for higher IPs. The EAs do not show this behavior. Accordingly, we would expect the rSCAN to be most reliable for calculating VBOs of materials with similar IPs. For the Si:TiO_2_ case, the VBO at the PBE level is already close to the *GW* value, and rSCAN retains this good agreement (Figure 3). Taking also the EA and the wider data set in Figure 4 into consideration, we conclude that rSCAN will provide more accurate offsets than PBE, but a higher level of theory like *GW* is required for <∼0.5 eV accuracy.

### 3.5 Band Alignments from Heterointerface Calculations

We now present the band offsets based on the heterointerface alignment approach, for the models shown in Figure 1. Models 1 and 2 are both based on the anatase (001) surface but differ in the type of bonds formed. Specifically, Model 1 has O atoms which form Si–O–Ti bridges, as well as atoms forming Si–O–Si bridges at the terminated Si surface. By contrast, all O atoms in Model 2 are 3-fold coordinated, either to two Ti and one Si atom, or vice versa. Model 2 is calculated to be more stable than Model 1 by 17 meV (PBE) or 144 meV (rSCAN), per surface Si atom. To investigate the influence of a wider range of bonding types, we generated Model 3. Although this model has been relaxed such that all atomic forces are zero, thermodynamically it is highly unstable (+0.9 eV/Si atom compared to Model 2, using PBE). Based upon these calculated energies, of the models involving the anatase (001) surface we might conclude that Model 2 is the most likely to be observed experimentally. However, we note that the energy of Model 1 is only slightly higher, and also that we have explored a very limited region of the phase space of potential interface structures. Model 4 is based on the (101) surface and contains a mix of 2-fold and 3-fold coordinated O atoms.

**Figure 5:**
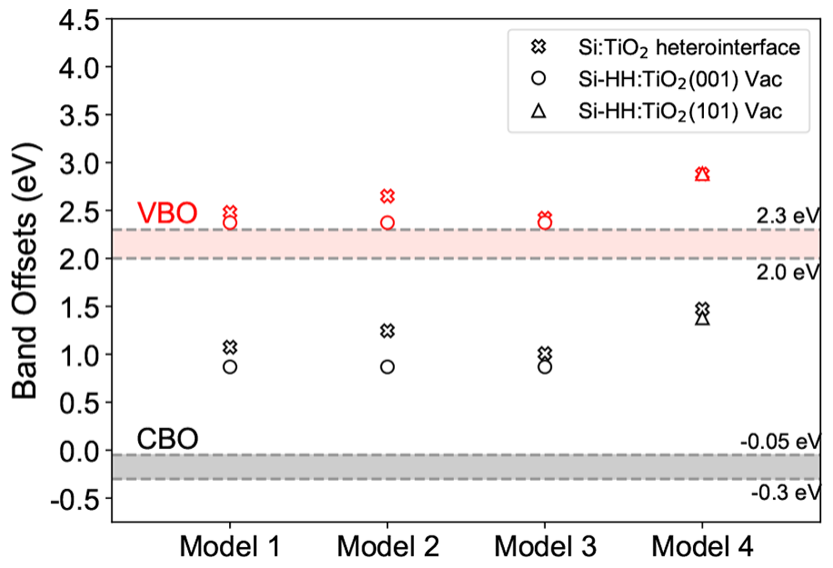
Calculated band offsets of Models 1–4 using heterointerface calculations along with the band offsets of Si-HH:TiO_2_(001) and Si-HH:TiO_2_(101) obtained using vacuum level alignments.

Figure 5 shows the band offsets calculated for these models, using PBE. First, we note that the values are similar (to within 0.4 eV) to those calculated for the H-terminated Si surface with the vacuum alignment method, but not similar to the O-terminated slabs, which are ∼2 eV lower (Figure 2). Therefore, even though the models 1–4 are based on O-terminated slabs, the O-layer does not have the same dramatic effect when the TiO_2_ slab is explicitly included. More generally, the agreement between the vacuum and heterostructure aligned offsets provides some justification for the experimental literature using the former to produce band diagrams.^25,26,43^

Considering models 1–3, we see how the presence of different bonding types introduces variations of order 0.24 eV in the band offsets. However, despite the unusual bonding present in Model 3, its band offsets are similar (to within 0.07 eV) to the much more ordered Model 1. A stronger effect can be seen for model 4, based on anatase (101), whose band offsets are 0.40 eV higher than those of model 1. This tracks the behavior observed for the vacuum alignment calculations (Figure 2), showing the importance of surface orientation.

Models 1–4 were constructed using the relaxed Si lattice parameters, which led to the TiO_2_ slab being put under strain. The lateral strains were +1.7% for the (001) surface and +4.8 and −2.0% for (101) in the two perpendicular directions, respectively. We also carried out calculations using the TiO_2_ lattice parameters, which led to an increase and decrease in the VBO and CBO respectively of order 0.1 eV (Supporting Information). We also recalculated the vacuum level alignment for the (101) surface when the TiO_2_ slab was placed under the above 4.8 and 2.0% strains, which led to increases in the band offsets by 0.3 eV.

**Figure 6:**
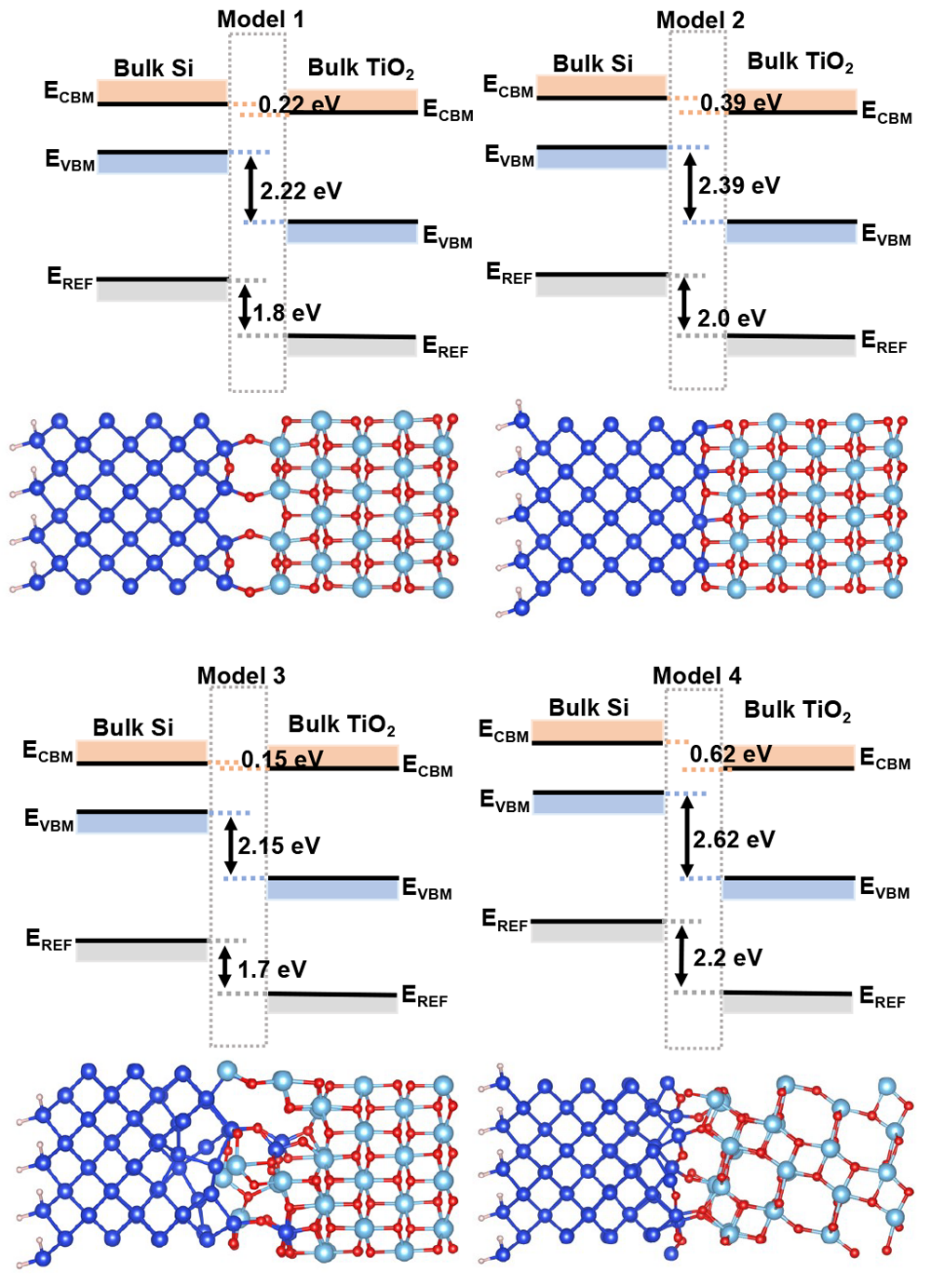
Heterostructure band offsets of Models 1–4 calculated by applying *GW* corrections as a postprocessing step. *E*_REF_ is the reference potential used in the alignment.

Finally, in Figure 6 we show the result of applying the *GW* corrections discussed in Sec. 3.3 to the PBE band edges. For the (001)-derived structures, the VBOs fall in the range 2.1–2.4 eV, while the CBOs are in the range 0.1–0.4 eV, forming a type-II alignment. Again, we note the similarity to the VBO/CBO values of 2.11 and 0.02 eV (Figure 3), obtained when *GW*-corrections were applied to the vacuum-aligned offsets.

## 4 Discussion

Considering first the VBOs, for almost all interface models considered, we find sizable valence band offsets of order 2 eV. The only calculation which predicted significantly smaller VBOs was the vacuum alignments with O-terminated Si. However, these large differences were not present in the heterointerface calculations (which also use O-terminated slabs), showing that the TiO_2_ overlayer effectively screens the large surface dipole of the O-terminated Si.

The VBOs were found to be largely independent of the calculation method used, showing an insensitivity to the Hubbard *U* value, the use of PBE or rSCAN, and even whether or not a postprocessing *GW* correction was applied. Applying corrections based on hybrid functionals did increase the VBO, however, with these corrections being sensitive to the fraction of exact exchange employed. The VBOs that we have calculated will act as barriers which prevent hole transport from the Si into the TiO_2_, as required to form electron selective contacts.

Now considering the CBOs, it is first important to note that they are more sensitive to the calculation method. Including a Hubbard *U* correction and varying *U* between 0–8 eV reduces the CBO from 1 to 0 eV. Applying postprocessing corrections also reduces the CBO from its PBE value, again by approximately 1 eV in the case of *GW*. There is, however, reasonable consistency with the CBOs calculated with the heterostructure or vacuum alignment methods. Taking the *GW*-corrected values as the most accurate gives CBOs of in the range 0.0–0.4 eV in vacuum-level or heterointerface alignment (Figures 2 and 5, and Supporting Information).

It is worth emphasizing that a positive CBO, as calculated here, would correspond to a type II heterojunction, while previous literature has reported degenerate conduction bands or a slightly negative CBO (type I), with type II alignment measured for structures including interlayers (Section 3.1). The difference between our calculated CBO and zero is small enough to fall within the error bars of our method, particularly given the highly idealized model of the interface that we have constructed. However, the fact that we calculate such similar positions for the conduction band edges of Si and TiO_2_ does show that—unlike the very large VBO—small changes in the relative band positions can enable or prevent carrier transport (electrons) across the interface. Our calculations have shown how the formation of different types of bonds (2-fold or 3-fold coordinated O), different anatase surface orientations, or application of strain, can account for this shift. Therefore, we do expect the electron transport properties to be sensitive to the fabrication method. However, forming a disordered array of bonds as in Model 3 does not lead to increased band offsets compared to the highly ordered models 1 and 2.

In terms of methodology, our benchmark calculations agree with a recent study,^59^ that rSCAN provides band offsets that are closer than PBE to experiment and to higher-level calculations. However, the rSCAN offsets are still not quantitative, and applying *GW* corrections to PBE in a postprocessing step is a simple alternative. An interesting avenue to explore would be to return to the calculation of bulk band edge corrections^50,52,53,58^ but using rSCAN as the starting point for the *GW* calculations. This would allow us to apply corrections as in Figure 3 to the rSCAN band edges.

Finally, returning to Si:TiO_2_, we have focused this first study on the anatase phase. Our calculations on model 3 provide some indication that our results may be transferable to an amorphous interface, but more work is required in order to confirm this. Also, in this study we have focused on slightly O-rich interfaces, but our calculations have not considered O-deficient interfaces. Generation and characterization of interface models based on TiO layers would provide insight into their properties as hole selective contacts.^16^

## 5 Conclusions

In conclusion, we have calculated the band offsets of the Si:anatase TiO_2_ interface from first-principles using two different methods—vacuum level and heterointerface alignment—investigating the role of atomistic structure and surface orientation. We have shown that, with the exception of vacuum calculations on O-terminated slabs, the results using different interface models and methods are broadly consistent with each other. Furthermore, treating exchange and correlation at the GGA, meta-GGA, or GGA+*U* levels of theory yields similar values of the valence band offset (VBO), but the conduction band offset (CBO) is much more sensitive, particularly to the *U* value. Applying postprocessing corrections based on hybrid functionals increases the VBO to values much larger than previous experimental reports, but using the *GW* approximation to calculate these corrections produces VBOs and CBOs which are closest to these experiments. We have also assessed the reliability of the rSCAN meta-GGA functional for calculating vacuum level alignments and found improvement compared to GGA calculations, but the numerical error is still of order 0.5 eV. We suggest that applying postprocessing *GW* corrections on top of GGA calculations represents the best balance between accuracy and computational efficiency as a method to calculate band offsets for this interface. The calculations show a large VBO to prevent hole transport from Si to TiO_2_, and a small, type-II CBO. Furthermore, we have shown that changes in the structure and orientation at the interface induces changes in the band offsets which are of the same size as the CBO itself. Therefore, it is likely that the preparation conditions will have a large effect on electron transport across the interface, due to the induced changes in the CBO.

## Supporting Information Available

Calculated IP and EA of Si and TiO_2_ slabs using PBE and rSCAN; computational details and results for rSCAN benchmarks; band alignments of interface models constructed using TiO_2_ lattice constants; band offsets of interface models using corrected band edge positions; and optimized slabs and interface models using PBE (PDF)
Optimized structures of Si slabs with various termination groups, TiO_2_(001) and TiO_2_(101) slabs, and heterointerface Models 1–4 in CASTEP cell format (ZIP)
