# Supplementary material for: First-Principles Band Alignments at the Si:Anatase TiO2 Interface
Source: ACS Omega. 2023 May 22;8(22):20138–47. doi: 10.1021/acsomega.3c02865 (PMC10249118; doi:10.1021/acsomega.3c02865)
Supplement: Supplementary file 1 — ao3c02865_si_001.pdf [file ao3c02865_si_001.pdf]

Supporting information: First-principles band alignments at the Si:anatase TiO_2 interface

Yide Chang, Jonathan R. Yates, and Christopher E. Patrick*

Department of Materials, University of Oxford, Parks Road, Oxford OX1 3PH, U.K.

E-mail: christopher.patrick@materials.ox.ac.uk

Section S1 provides the calculated IP and EA of Si with different termination groups (Table S1) and anatase TiO_2(001) and (101) slabs (Table S2). In Section S2, the computational methodology for the rSCAN benchmark calculations is explained, and Table S3 presents the results of these calculations, including lattice constants, IP, EA, and band gaps. Section S3 discusses the calculated band alignments of Si:TiO_2 heterointerfaces, constructed with TiO_2 lattice constants using PBE (Figure S1). Section S4 provides the calculated band offsets of the heterointerface Models 1-4 (Table S4) using corrected band edges obtained from PBE0, mPBE0, and the *GW* approximation. Optimized structures of Si slabs with various termination groups, TiO_2(001) and TiO_2(101) slabs, and heterointerface Models 1-4 are provided separately in CASTEP cell format.

Section S1 Calculated IP and EA of Si and TiO_2 slabs

Table S1: Calculated IP and EA (in eV) of Si slabs with different termination groups using PBE and rSCAN

	Si termination group				
PBE	HH	OH	OO	Si(001)	Si(111)
IP	4.60	7.11	7.12	4.92	4.80
EA	3.99	6.50	6.51	4.31	4.19
rSCAN					
IP	4.80	7.34	7.27	5.16	5.00
EA	4.03	6.57	6.50	4.39	4.24

Table S2: Calculated IP and EA (in eV) of anatase TiO_2(001) and (101) slabs using PBE and rSCAN

	PBE + U					rSCAN
TiO_2(001)	0	2	4	6	8	
IP	6.97	6.91	7.02	7.01	6.98	7.20
EA	4.85	4.68	4.61	4.33	3.89	4.65
TiO_2(101)						
IP	7.43	-	-	-	-	7.91
EA	5.31	-	-	-	-	5.37

S2 Computational detail and results of 21 semiconductors using PBE and rSCAN

Table S3: Calculated lattice constants (a and c, in Å), band gaps (E_g), IP, and EA in eV using PBE and rSCAN

	PBE					rSCAN				
	a	c	E_g	IP	EA	a	c	E_g	IP	EA
BN	3.624	-	4.46	6.99	2.53	3.607	-	5.00	7.23	2.23
C	3.571	-	4.13	5.56	1.44	3.554	-	4.37	5.71	1.34
Si	5.468	-	0.61	4.81	4.20	5.439	-	0.77	5.01	4.25
Ge	5.762	-	0.00	4.36	4.36	5.682	-	0.29	4.83	4.54
AlP	5.505	-	1.63	5.70	4.07	5.473	-	1.91	5.97	4.05
AlAs	5.730	-	1.51	5.30	3.79	5.679	-	1.80	5.54	3.74
AlSb	6.227	-	1.23	4.78	3.54	6.179	-	1.51	5.11	3.60
GaP	5.505	-	1.58	5.44	3.86	5.451	-	1.91	5.58	3.67
GaAs	5.748	-	0.16	4.91	4.75	5.673	-	0.91	5.15	4.24
GaSb	6.213	-	0.00	4.48	4.48	6.139	-	0.35	4.71	4.36
InP	5.956	-	0.44	5.12	4.68	5.902	-	1.00	5.42	4.43
InAs	6.191	-	0.00	4.77	4.77	6.108	-	0.03	5.10	5.07
InSb	6.632	-	0.00	4.47	4.47	6.564	-	0.00	4.72	4.72
ZnS	5.443	-	2.01	5.98	3.97	5.382	-	2.69	6.35	3.66
ZnSe	5.734	-	1.15	5.63	4.49	5.668	-	1.84	5.93	4.10
ZnTe	6.186	-	1.09	5.10	4.01	6.125	-	1.75	5.39	3.64
CdS	5.928	-	1.03	5.92	4.89	5.867	-	1.48	6.32	4.84
CdSe	6.196	-	0.49	5.61	5.12	6.123	-	0.97	6.16	5.19
CdTe	6.624	-	0.59	5.20	4.61	6.568	-	1.05	5.69	4.63
ZnO	3.282	5.296	0.72	6.02	5.30	3.233	5.211	1.21	6.17	4.95
GaN	3.217	5.243	1.72	5.78	4.06	3.180	5.182	2.36	5.97	3.61

To gain a more comprehensive understanding of the performance of rSCAN and how it compares to PBE in predicting the properties of various materials, we have calculated the band gap, IP, and EA of various materials using PBE and rSCAN functionals. Specifically, we focused on GaN and ZnO in the wurtzite structure, C, Si, and Ge in the diamond structure, and BN, AlP, AlAs, AlSb, Gap, GaAs, GaSb, InP, InAs, InSb, ZnS, ZnSe, ZnTe, CdS, CdSe, and CdTe in the zinc-blende structure.

A plane wave cut-off energy of 700 eV and 8×8×8 k points are used in PBE bulk calculations except for CdS, CdSe, and CdTe. An 800 eV plane wave cut-off and 8×8×8 k points are used in RSCAN bulk calculations except for CdS, CdSe, and CdTe. For slab

calculations, a 6×6×1 k points grid is used, and the plane wave cut-off remains the same. When calculating properties of CdS, CdSe, and CdTe, a plane wave cut-off energy of 900 eV and 1000 eV are used for PBE and RSCAN calculations, respectively. Relaxed lattice parameters are used for all calculations.

S3 Calculated band alignments of Model 1 and 2 with TiO_2 lattice constants

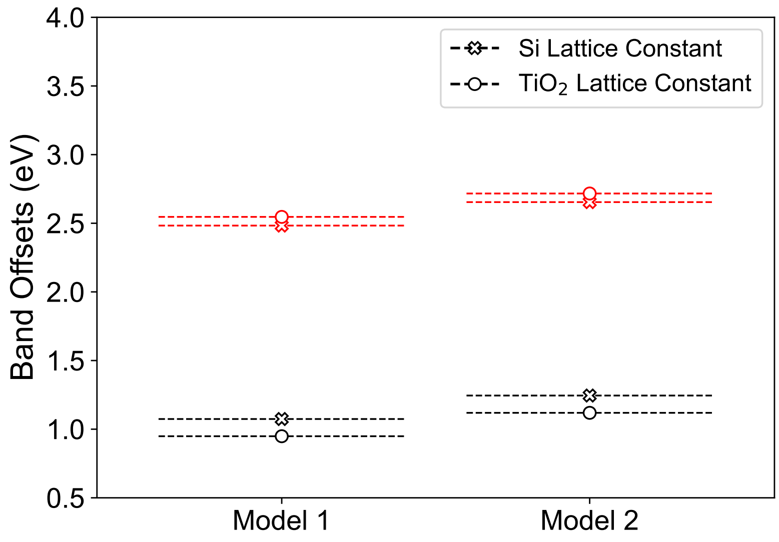

Figure S1: Calculated VBOs (red dotted line) and CBOs (black dotted line) of Si:TiO_2(001) interface models constructed using in-plane lattice parameters of Si and TiO_2.

The impact of lattice mismatch on the band alignments of the Si:TiO_2(001) interface model was investigated by constructing two interface models (Model 1 and Model 2) using the in-plane lattice parameters of TiO_2(001) instead of those of Si. The resulting valence band offsets (VBOs) and conduction band offsets (CBOs) are compared with those of previous interface models constructed using the Si lattice constants, as shown in Figure S1. The calculations reveal that the interface models with in-plane lattice parameters of TiO_2 have

higher VBOs than those with Si lattice constants, with an increase of approximately 0.07 eV when using PBE. Furthermore, the interface models with in-plane lattice parameters of TiO_2 exhibit lower CBOs, with a decrease of 0.13 eV. These findings demonstrate that different lattice mismatches impact the band alignments of the Si:TiO_2 interface model, quantifying the importance of strain.

S4 Corrected band offsets of Model 1-4 using PBE0, mPBE0, and *GW* approximation

Table S4: Calculated VBOs and CBOs of Si:TiO_2 interface models using corrected band edges obtained from PBE0, mPBE0, and the *GW* approximation, in eV.

	Si:TiO_2 interface models			
PBE0	Model 1	Model 2	Model 3	Model 4
VBOS	3.23	3.40	3.17	3.63
CBOs	0.68	0.86	0.62	1.08
mPBE0				
VBOs	3.16	3.33	3.10	3.56
CBOs	0.69	0.87	0.63	1.09
GW				
VBOs	2.22	2.39	2.15	2.62
CBOs	0.22	0.39	0.15	0.62
